# The Silkworm (*Bombyx mori*) Neuropeptide Orcokinin’s Efficiency in Whitening and Skincare

**DOI:** 10.3390/ijms26030961

**Published:** 2025-01-23

**Authors:** Pingyang Wang, Xiao Xiao, Ya Yang, Guiqiu Liang, Shengtao Lu, Liang Tang, Hongyan Huang, Ji He, Xiaoling Tong

**Affiliations:** 1 State Key Laboratory of Resource Insects, College of Sericulture, Textile and Biomass Sciences, Southwest University, Chongqing 400715, China; wangpingyang.yczg@163.com; 2 Guangxi Key Laboratory of Sericultural Genetic Improvement and Efficient Breeding, Guangxi Research Academy of Sericultural Science, Guangxi Sericultural Technology Promotion Station, Nanning 530007, China; xiaoxiao1991627@163.com (X.X.); liangguiqiu0816@163.com (G.L.); ty008tl@163.com (L.T.); gxhhy@163.com (H.H.); 17777190006@163.com (J.H.); 3 College of Sports and Health Sciences, Guangxi University for Nationalities, Nanning 530006, China; yangyaguangximinda@163.com; 4 College of Agriculture, Guangxi University, Nanning 530004, China; 17607789032@163.com

**Keywords:** Orcokinin, whitening, tyrosinase, melanin, neuropeptides

## Abstract

The silkworm neuropeptide Orcokinin (abbreviated as BommoOK) is equipped with multiple biological functions, one of which acts as a pigmentation inhibitor. To explore the whitening efficiency of BommoOK, the inhibitory effects on tyrosinase and its adaptability on the cell for six mature peptides of BommoOK were investigated in this paper. At the same time, BommoOKA_type4, the peptide with the best melanin inhibition effect, was used as an additive to prepare a whitening cream, and the effects on skin moisture, oil content, fine lines, skin glossiness, pores, and pigment depth were determined. The results revealed that the cream added with BommoOKA_type4 peptide showed a good improvement effect on the skin, especially in significantly reducing the pigment depths of skin. The results displayed a potential application of BommoOK in whitening and skincare products as an excellent additive and provide certain references for the mechanism research of BommoOK in inhibiting melanin synthesis.

## 1. Introduction

Neuropeptide Orcokinin (abbreviated as OK) belongs to a new class of neuropeptide and is widely present in Arthropoda [1]. OK was named a kinin because of the myotropic character of the hormone [2], and because it is mainly found in Crustacea [3,4] and Insecta [5], as well as in other Arthropoda like Entognatha and Arachnida. Additionally, *OK* was also found in Nematoda, Tardigrada, and Priapulida [6]. *OK* was first identified from *Bombyx mori* in 2011 and charactered no myotropic properties like other insects [5,7]. The gene encoding the OK neuropeptide typically has two transcripts, *OKA* and *OKB*, which are either encoded by two separate genes or formed by alternative splicing from the same gene [8,9,10]. In Insecta, two *OK* transcripts are produced by alternative splicing [11]. In *B. mori*, two transcripts, *BommoOKA* and *BommoOKB,* are formed through alternative splicing, of which *BommoOKA* is characterized by more structural subtypes [12]. The final mature products encoded by the *OK* gene are neuropeptides, including OKA and OKB [10,13]. Different transcripts were translated into their own protein precursors and cleaved into a variety of mature peptides, including signal peptides, various types of functional peptides, and multiple repeats of the same functional peptide, etc. [10,14]. Five mature peptides were detected in *B. mori* [7].

*OK* is mainly expressed in the ventral nerve cord and other endocrine nerve cells, central nerve cells, the prothoracic gland, and midgut endocrine cells [7]. In situ hybridization results in *B. mori* showed that *BommoOK* is mainly present in the ventral ganglion, central nervous system and midgut endocrine cells. The expression level of *BommoOKA* is lower in molting than in other stages, and this trend is inversed in the case of *BommoOKB* [7,12].

The structure of *OK*s is complex and diverse, and their functions are also varied, primarily participating in the regulation of motor nerves and other physiological processes [6]. In *Orconectes limosus*, an irritation test was performed after immersing the hindgut in vitro in polypeptide solution, and the results showed that *OK* can enhance the peristalsis of the hindgut in vitro [2]. Electrophysiological studies revealed their role in the excitability of the central nervous system circuits [3,15]. In *Leucophaea maderae*, an injection of OK peptide into accessory medulla suggested that OK-related peptides play an important role in the light entrainment pathways of the circadian clock [16]. Subsequent RNAi studies in *T. castaneum* showed that *OK* plays an important role in awaking behavior and regulation of the circadian rhythm [10]. Similarly, RNAi studies on *Blattella germanica* showed that *OK* is involved in the regulation of vitellogenin expression in body fat, and the effect was not dependent on the juvenile hormone [1]. Another RNAi research study into *R. prolixus* suggested that *OK* plays a role in molting regulation [11,17]. In *B. mori*, BRFa (Bommo-FMRFamides) binds to its receptor BMSR (Bommo-myosuppressin receptor) to regulate ecdysone synthesis by adjusting the cAMP content. As a prothoracicotropic factor, OK can bind to an unknown receptor to influence the biosynthesis of ecdysone by weakening the enhancing effect of BRFa on ecdysone synthesis through the cAMP pathway [7,18].

Melanin is a polymer similar to polyphenols, with complex structures and colors, widely distributed in bacteria, fungi, plants, and animals [19]. As a protective mechanism, melanin was synthesized in melanocytes when exposed to ultraviolet radiation, and transported from the basal layer to the differentiated keratinocytes of upper dermis. Then, they were combined with the movement protein and dynein of cytoskeletal to form a special super-nucleocapsid structure [20,21]. However, melanin does not rapidly dissipate after the cessation of ultraviolet radiation [22], which leads to pigmentation like chloasma and freckles. Tyrosinase, the first enzyme in the melanin synthesis pathway, is the rate-limiting enzyme [20,23]. Therefore, as whitening agents in skincare products, most of which are tyrosinase inhibitors, their inhibitory activity on tyrosinase shows the whitening effect [20]. Common whitening agents are these organic compounds and their derivatives, including resorcinol, ascorbic acid, salicylic acid, α-hydroxy acids, arbutin, glycyrrhizin, tranexamic acid, niacinamide, kojic acid, resveratrol, bisabolol, acetyl glucosamine, etc., [24]. Ascorbic acid, also known as vitamin C, can inhibit the tyrosinase activity through its antioxidant properties, and help to reduce and inhibit the production of melanin by reducing dopa and dopaquinone [25]. Arbutin, a derivative of hydroquinone, can prevent the conversion of L-tyrosine to L-dopa catalyzed by tyrosinase to achieve a whitening effect by inhibiting the tyrosinase activity [26]. After comparison with three other tyrosinase inhibitors (hydroquinone, arbutin, and kojic acid), 4-Butylresorcinol is the most efficient one, showing high application value [27].

In our previous studies, the CRISPR/cas9 tool and mature peptide subcutaneous injection were used to character the function of *BommoOK*, which demonstrated an inhibitory effect on pigmentation in *B. mori*. Further identification revealed that one of the mature peptides, BommoOKA_type2, is the key for pigmentation inhibition [28]. To investigate the inhibitory effect on tyrosinase, the adaptability to the skin, and the whitening effect on the skin for BommoOK mature peptides, the inhibitory rate of six mature peptides on tyrosinase was measured. Then, a UV-irradiated guinea pig (*Cavia porcellus*) model was constructed to explore the adaptability and whitening effect of these peptides, and a skincare product was prepared using peptides as additives. A skin analysis instrument was employed to analyze the relevant evaluation indicators for the skin after use of the product. Therefore, we systematically explored the effect of these peptides in whitening skincare in this study, which provides experimental evidence for the mechanism of peptides in inhibiting pigmentation and product development.

## 2. Results

### 2.1. Orcokinin Has an Inhibitory Effect on Tyrosinase

By detecting the inhibitory rate of peptides on tyrosinase, it was found that BommoOKA_type3, BommoOKA_type4, and BommoOKB_type1 had a certain inhibitory effect on tyrosinase. The reaction was still ongoing after 60 s of reaction and continued to 180 s, while the negative control without any inhibitors was already completed at 60 s. BommoOKA_type2 and BommoOKA_type5 had no effect on tyrosinase that also completed around 60 s, while BommoOKA_type1 seemed to tend to accelerate tyrosinase, with an OD value less than 0.1 at 10 s (Table 1).

**Figure 1:**
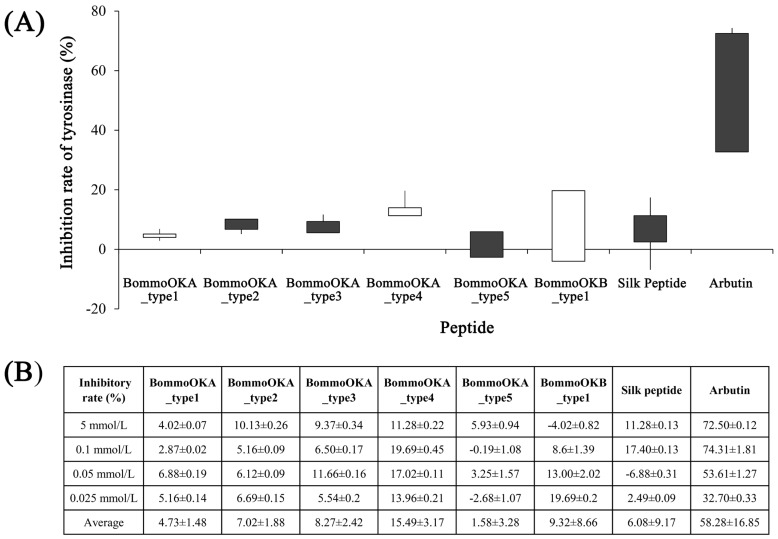
The mature peptide of *BommoOK* has a certain inhibitory rate on tyrosinase. (**A**) Stock chart of the inhibitory rate of different concentrations of *BommoOK* mature peptide on tyrosinase. (**B**) Table of the inhibitory rate of different concentrations of BommoOK mature peptide on tyrosinase. Silk peptide here represents the hydrolysate of silk, which is always used as the additive of whitening and skincare products.

The inhibition rate of peptides on tyrosinase was calculated using the classical calculation formula [29], when the reaction proceeded to the fifth minute. It was found that all peptides had a certain inhibitory effect on tyrosinase and showed a trend of increasing and then decreasing with the decrease in concentration. BommoOKA_type1, BommoOKA_type4, and BommoOKB_type1 exhibited relatively high inhibition rates at lower concentrations, while other peptides at moderate concentrations (Figure 1A). In addition to the positive control Arbutin, the inhibition rates of BommoOKA_type4 (all concentrations), BommoOKB_type1 (0.05 mmol/L and 0.025 mmol/L), BommoOKA_type3 (0.05 mmol/L), and BommoOKA_type2 (5 mmol/L) were greater than 10%. BommoOKA_type4 (0.1 mmol/L) and BommoOKB_type1 (0.025 mmol/L) had the highest inhibition rate of 19.69%, both of which were higher than the highest inhibition rate (17.4%) of silk peptide (Figure 1B). Based on the average inhibition rate, the inhibition rate rank of several peptides was BommoOKA_type4 > BommoOKB_type1 > (BommoOKA_type3, BommoOKA_type2, BommoOKA_type1) > BommoOKA_type5.

### 2.2. Skin Adaptability Test of Orcokinin Mature Peptide

**Figure 2:**
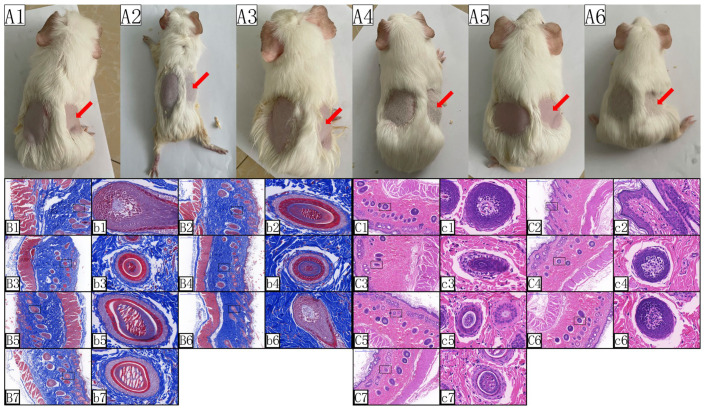
Exploration of the application of OK mature peptide in skin whitening and care. (**A**) Guinea pig model, with a blank control on the left side of the back and a peptide solution applied on the right side. A1 to A6 represent BommoOKA_type1, BommoOKA_type2, BommoOKA_type3, BommoOKA_type4, BommoOKA_type5, and BommoOKB_type1, respectively. The red arrows represent a peptide solution applied on the right side of the back; (**B**) represents the results of Masson’s trichrome staining. B1 to B6 represent the application of BommoOKA_type1, BommoOKA_type2, BommoOKA_type3, BommoOKA_type4, BommoOKA_type5, and BommoOKB_type1 peptide solutions, respectively. B7 represents the blank control, and b1 to b7 are local magnifications of B1 to B7, respectively; (**C**) represents the results of HE staining. C1 to C6 represent the application of BommoOKA_type1, BommoOKA_type2, BommoOKA_type3, BommoOKA_type4, BommoOKA_type5, and BommoOKB_type1 peptide solutions, respectively. C7 represents the blank control, and c1 to c7 are local magnifications of C1 to C7, respectively. (**B**,**C**) scale bar: 500 μm; (**b**,**c**) scale bar: 50 μm.

The UV-irradiated areas of skin became rough and hardened, lost elasticity, and showed signs of skin aging after UV irradiation. There was no significant difference between the treated group and the control group (Figure 2A) by visual inspection. Masson staining and HE staining of pathological tissues showed that melanocytes and melanin granules increased in the control group, with epidermal hyperplasia, thicker stratum corneum and stratum granulosum, loosely arranged tissues, and the presence of inflammatory cells in the dermis. Many inflammatory cells were detected from the treatment groups of BommoOKA_type2 and BommoOKA_type3, while significant epidermal hyperplasia was detected from that of BommoOKB_type1, and loosely arranged tissues from that of BommoOKA_type1. In comparison, the stratum granulosum was significantly thinner in the treatment group of BommoOKA_type3, while inflammatory factors were reduced, and epidermal hyperplasia improved in the treatment groups of BommoOKA_type4 and BommoOKA_type5. From the perspective of melanin inhibition, many stretched melanin granules emerged in all control groups and the treatment groups of BommoOKA_type1, while the melanocyte decreased in the treatment groups of BommoOKA_type4 and BommoOKA_type5 (Figure 2B,C). In summary, the inhibitory strength of six BommoOKs was (BommoOKA_type4, BommoOKA_type5) > BommoOKB_type1 > (BommoOKA_type1, BommoOKA_type2, BommoOKA_type3). Obviously, BommoOKA_type4 and BommoOKA_type5 hold better melanin inhibition effects with almost no damage to tissues (Figure 2).

### 2.3. Skincare Product and Whitening Effects

**Figure 3:**
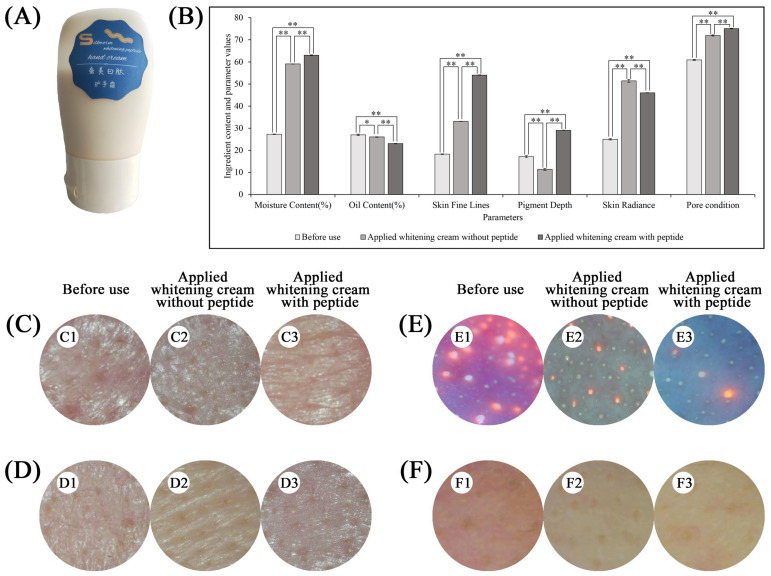
Whitening cream prepared with BommoOKA_type4 as an additive. (**A**) Whitening cream product style; (**B**) Evaluation of the use of whitening cream, * represents a *t*-test result with 0.01 ≤ *p* < 0.05 while ** represents a *t*-test result with *p* < 0.01; (**C**) Skin condition of cheeks before and after using whitening cream; (**D**) The condition of the forehead skin before and after the use of whitening cream; (**E**) UV detection of skin pores at the nasal wing; (**F**) Infrared detection of skin capillaries on the cheeks. (**C1**–**C3**,**D1**–**D3**,**E1**–**E3**,**F1**–**F3**), respectively, represent before use, 1 h after using cream without added peptides, and 1 h after using cream with added peptides.

Through tyrosinase inhibition and skin adaptability testing, it was found that BommoOKA_type4 not only had better tyrosinase inhibition (19.69%), but also skin adaptability (reduced inflammatory factor, improved epidermal proliferation, and decreased melanocytes). Therefore, BommoOKA_type4 was selected as an additive to prepare a whitening cream (Figure 3A), which was white and milky with a delicate and smooth touch.

After applying the product to cheeks (Figure 3C) and forehead (Figure 3D) for 1 h, a skin analyzer was employed to evaluate the skin condition before and after use of the product. The results showed that hydration, oil leakage, fine lines, color, and pore of skin were all improved, whether the product contained peptides or not, but was better than no use of the skincare product (Figure 3B–F). It had better effects on improving hydration and oil leakage, fine lines, and pores than that without the BommoOKA_type4 peptide. In addition, it was noteworthy that there was no effect on pigmentation for the cream without the peptide, while it could significantly improve the skin pigmentation after adding the peptide into the cream. These results above indicated that the peptides BommoOKA_type4 indeed had positive effects on whitening (Figure 3B–F).

## 3. Discussion

Neuropeptides with biological functions were called neuropeptides if they were secreted by neural tissues and involved in nervous system functions, which were characterized by low content, high activity, and extensive and complex functions (including food intake regulation, improved metabolism, neural regulation and reproduction, etc.) [30]. However, there have been few reports on the pigmentation regulation of neuropeptide. One important function of *BommoOK*, as a multifunctional neuropeptide, was pigmentation regulation. In silkworm *q-l* (quail like) mutants, *BommoOK* was a losing function, and as a result, many quail-like spots appeared while the corresponding wild-type silkworms were white silkworms [6,28], which provided evidence of a pigmentation regulation function of *BommoOK*. Pigmentation is the process of melanin production, transport, and storage in an organism, which is one of their protective mechanisms. Tyrosinase is the key rate-limiting enzyme for melanin production and has become a target for screening whitening agents or treating diseases related to abnormal pigment metabolism [31]. Many tyrosinase inhibitors have been identified, among which arbutin is a commonly used tyrosinase inhibitor in skincare products, and its whitening effect can even last for more than 8 weeks [32]. By measuring the inhibitory effect of BommoOK on tyrosinase, it was found that the mature peptides have a certain inhibitory effect, but the inhibition rate is not ideal. Compared with the inhibition rate of more than 70% for β-arbutin, the inhibition rate of BommoOK is about 20%, which is relatively low. An effective way to improve the inhibition rate was modifying the structure of the peptide. The structure of OK mature peptide was conservative on N-term. After a terminal deletion analysis of O. limosus, OK (NFDEIDRSGFGFN) was performed; the results indicated that removal of the N-terminal asparagine resulted in a minor reduction in activity (93.6% of OK). But after removal of the first two to four N-terminal amino acids, it led to a complete loss of activity. As a comparison with C-terminal deletion analysis, it substantially reduced activity after the first one to three amino acids were removed and produced a completely inactive analog when more amino acids were removed [33]. Based on these facts above, the N-terminal of OK might closely relate to the functional domain while the N-terminal of OK might closely relate to the binding domain. As an example, deoxyarbutin, a product of structural modification from β-arbutin, hold more than 350 times the whitening effect of β-arbutin [32]. In addition, tyrosinase is the only first enzyme in the melanin synthesis pathway [34], and there are many other possible targets in the pathway to develop whitening skincare products.

As an additive of skincare products, skin adaptability analysis was necessary to explore the rejection reaction and appropriate addition concentration. It may not achieve an ideal whitening effect with a lower concentration, and conversely it may harm skin health with a higher concentration. The typical concentration of β-arbutin in skincare products is 3% [32]. A novel peptide Nigrocin-OA27, derived from *Odorrana andersonii*, has been shown to significantly reduce melanin content, which not only holds a significant whitening effect but also exhibits non-toxicity and antioxidant capacity. As an application, Nigrocin-OA27 has a curative effect on melasma by inhibiting abnormal melanin synthesis [35]. In addition, a peptide OA-VI12, from the skin of amphibians, exhibits anti-light damage activity. The effect of OA-VI12 on cell viability and melanin content with mouse melanoma cells (B16) was performed, and the results showed a significant inhibitory effect on melanogenesis without cytotoxicity [36]. Some mature peptides of *BommoOK* also showed a certain inhibitory effect on melanin without cytotoxicity. In addition, whitening cream was prepared using BommoOKA_type4, with the best inhibitory effect, as an additive, and the skin adaptability testing showed a good skin adaptability, significant improvement in skin condition, and importantly, better improvement in pigment depth. Next, the structure of these peptides, especially BommoOKA_type4, would be modified to achieve the best inhibitory effect and develop mature whitening products.

## 4. Materials and Methods

### 4.1. Peptide Preparation

**Figure 4:**
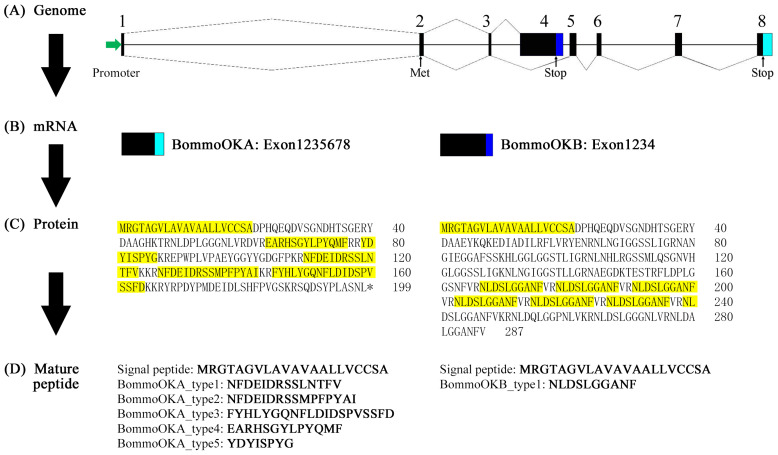
The fountainhead of BommoOK mature peptides. (**A**) Structure on genome level. The rectangles with different colors represent the exons, while the solid line represents the introns. The green arrow represents the promoter. The dashed line represents the manner of alternative splicing. (**B**) Structure on mRNA level. *BommoOKA* and *BommoOKB* are formed through alternative splicing. The numbers 1–8 represent the numbering of exons (**C**) Structure on protein level. The mature peptides are all marked with a yellow background. * represents the terminator codon. (**D**) Structure on peptide level.

The *BommoOK* has predicted 6 mature peptides in *B. mori* [7,28]. These peptides were named as BommoOKA_type1 (NFDEIDRSSLNTFV), BommoOKA_type2 (NFDEIDRSSMPFPYAI), BommoOKA_type3 (FYHLYGQNFLDIDSPVSSFD), BommoOKA_type4 (EARHSGYLPYQMF), BommoOKA_type5 (YDYISPYG), and BommoOKB_type1 (NLDSLGGANF), respectively, based on our previous study [28] in this study (Figure 4). The peptides were synthesized by GenScript Biotech Co., Ltd. (#SC1208; Piscataway, NJ, USA) with a purity greater than 95%, and verified by HPLC (high performance liquid chromatography) and MS (mass spectrum).

### 4.2. Tyrosinase Inhibition Rate Detection

According to the classical method of tyrosinase activity detection, the inhibitory effect of these peptides on tyrosinase inhibition was determined by employing β-arbutin as positive controls (Table 2) [29]. The absorbance of the reaction solution at 475 nm was measured at 10 s, 60 s, 120 s, and 180 s using a tyrosinase activity assay kit (Boxbio, Beijing, China), with three replicates for each sample. The peptide solution (5 mmol/L) was then diluted 50-fold (0.1 mmol/L), 100-fold (0.05 mmol/L), and 200-fold (0.025 mmol/L), respectively, and then the inhibitory rate was calculated after a 5 min reaction.

### 4.3. Skin Adaptability Testing

Model preparation: Six adult healthy guinea pigs with normal development were selected. The fur on both sides of the back was shaved to expose a circular area with a diameter of 4 cm. Ultraviolet (UV) irradiation was performed at 10 cm from the skin in the exposed area, once daily with a dose of 700 mJ/cm^2^, and continued for 7 consecutive days, with a cumulative dose of 5000 mJ/cm^2^. A metal halide lamp was used as the light source with a power of 1000 W and a peak spectrum of 360 nm, including 70% long-wave ultraviolet (UVA) and 4% medium-wave ultraviolet (UVB). After UV-irradiation treatment, the peptide solution (5 mmol/L) was painted to the right, and an equal amount of deionized water was applied to the left. Tissue sections and staining observations were performed after 24 h.

Sectioning: Approximately 0.4 cm^3^ of subcutaneous tissue from the drug administration area was excised, cleaned with physiological saline, and fixed. After dehydration (DIAPATH, Donatello, Milan, Italy) and hardening treatment, it was subsequently made transparent and subjected to a wax immersion treatment. After paraffin embedding (Wuhan Junjie Electronics Co., Ltd., JB-P5, Wuhan, China), the tissue was sectioned using a microtome (Shanghai Leica Instrument Co., Ltd., RM2016, Shanghai, China). The arranged sections were organized (Zhejiang Jinhua Kedi Instrument Equipment Co., Ltd., KD-P, Jinhua, China) for HE staining and Masson staining.

HE staining: The paraffin sections were placed in xylene I for 20 min, xylene II for 20 min, anhydrous ethanol I for 5 min, anhydrous ethanol II for 5 min, and 75% alcohol for 5 min for dewaxing treatment; after washing with water, they were placed into hematoxylin staining solution for 3–5 min, differentiated with differentiation solution after washing, and returned to blue with bluing solution after washing; after rinsing with running water, they were dehydrated with 85% and 95% alcohol solutions for 5 min each successively; after immersed into eosin staining solution for 5 min, they were placed in anhydrous ethanol I for 5 min, anhydrous ethanol II for 5 min, anhydrous ethanol III for 5 min, xylene I for 5 min, and xylene II for 5 min for transparency treatment. After, they were sealed with neutral gum, they were examined (Nikon Eclipse E100, Tokyo, Japan) and imaged (Nikon DS-U3, Tokyo, Japan) using a microscope.

Masson staining: The paraffin sections were placed in xylene I for 20 min, xylene II for 20 min, anhydrous ethanol I for 5 min, anhydrous ethanol II for 5 min, and 75% alcohol for 5 min for dewaxing treatment; after washing with water, they were immersed into Masson A solution overnight, washed with water, then immersed into a mixture of Masson B and Masson C for 1 min, washed with water, then differentiated using an alcohol solution containing 1% hydrochloric acid. Then, they were washed with water, then immersed into Masson D solution for 6 min, washed with water, then immersed into Masson E solution for 1 min, drained, and directly immersed into Masson F solution for 30 s. Subsequently, the sections were rinsed with 1% glacial acetic acid for differentiation, dehydrated with anhydrous ethanol three times for 5 min each, treated with xylene for transparency for 5 min, and then sealed with neutral gum. The sections were examined (Nikon Eclipse E100, Japan) and imaged (Nikon DS-U3, Japan) using a microscope.

### 4.4. Preparation of Whitening Cream and Effect Detection of Whitening Skincare

Preparation of peptide liposomes: A 0.6 mg/mL peptide solution was obtained by dissolving the peptide in PBS solution (pH = 4.7), and the peptide solution was equivolumetrically and dropwise added into the organic phase containing soy lecithin (2 mg/mL), cholesterol (0.1 mg/mL), and sodium cholate (0.1 mg/mL), while stirring continuously at 300–400 rpm. Then, the mixture was treated with 500 W ultrasound for 8 min and subjected to vacuum rotary evaporation at 37 °C. When a milky white film appeared, a small amount of Tween-80 and PBS buffer were added to shake and dissolve the film. Then, the mixture was dehydrated again by vacuum rotary evaporation at 37 °C, followed by 500 W ultrasound treatment for 4 min. Last, the peptide liposome powder, containing about 21% of the target peptide, was obtained by filtering through a 0.45 μm microporous membrane and freeze-dried.

Preparation of peptide whitening cream: The soothing anti-allergic agent (6.16%, final mass fraction, the same as below), plant extract (4.11%), and antioxidant (2.05%) were added to deionized water (48.13%), and heated to 50 °C with a continuous speed of 3000 rpm, during which xanthan gum (1.03%) was added. Then, the aqueous base was obtained. The natural oil (8.21%), emulsifier (6.16%), emulsifying agent (12.32%), and peptide liposome powder (3.21%) were mixed and heated to 80 °C, then the oil phase base was obtained. Continuously, the oil phase base and aqueous base were mixed and emulsified for 30 min with a speed of 4000 rpm, followed by cooling to 40 °C, during which the essence (2.05%), preservative (6.16%), and regulator (0.41%) were added in sequence, then homogenized for 40 min with a speed of 4000 rpm. Last of all, the peptide whitening cream was cooled to room temperature for later use.

Application effect detection of peptide whitening cream: Two adjacent areas (area a and b) of 2 × 2 cm^2^ were marked on the back of the hand. The Magic Mirror Smart Skin Analyzer (Guangzhou Magic Beauty Instrument Equipment Co., Ltd., Guangzhou, China) was used to detect the moisture and oil content of the skin, as well as fine lines, skin gloss, pigment depth, and pore condition, to evaluate the skin state. Then, one drop (about 0.05 mL) of peptide whitening cream and a whitening cream without peptides were applied to area a and b, respectively. One hour later, the Magic Mirror Smart Skin Analyzer was used again to detect the relevant indicators to analyze the role of peptides in whitening and skincare.

## 5. Conclusions

From the results of this study, we found that all BommoOK mature peptides had a certain inhibitory effect on tyrosinase, of which BommoOKA_type4 and BommoOKB_type1 held the highest inhibition rate, and the inhibition rate rank of several peptides was BommoOKA_type4 > BommoOKB_type1 > (BommoOKA_type3, BommoOKA_type2, BommoOKA_type1) > BommoOKA_type5. The skin adaptability test showed that BommoOKA_type4 and BommoOKA_type5 hold better melanin inhibition effects with almost no damage to tissues. After assessing the skincare product with BommoOKA_type4 selected as an additive, the peptides BommoOKA_type4 did indeed have positive effects on whitening.

## Figures and Tables

**Table 1:** The absorbance changes on tyrosinase reaction.

Grouping	Reagent	OD Value			
		10 s	60 s	120 s	180 s
Sample Background	PBS, Peptide	0.047 ± 0.003	-	-	0.047 ± 0.001
Reagent Background	PBS, Tyrosinase	0.042 ± 0.004	-	-	0.043 ± 0.004
Negative Control	PBS, Dopa, Tyrosinase	0.121 ± 0.035	0.052 ± 0.041	0.046 ± 0.009	0.043 ± 0.001
Positive Control	PBS, Dopa, Tyrosinase, β-Arbutin	0.125 ± 0.004	-	-	0.041 ± 0.005
BommoOKA_type1	PBS, Dopa, Tyrosinase, BommoOKA_type1	0.082 ± 0.052	0.043 ± 0.040	0.044 ± 0.004	0.043 ± 0.002
BommoOKA_type2	PBS, Dopa, Tyrosinase, BommoOKA_type2	0.138 ± 0.044	0.085 ± 0.015	0.059 ± 0.013	0.064 ± 0.050
BommoOKA_type3	PBS, Dopa, Tyrosinase, BommoOKA_type3	0.156 ± 0.056	0.103 ± 0.030	0.105 ± 0.023	0.042 ± 0.001
BommoOKA_type4	PBS, Dopa, Tyrosinase, BommoOKA_type4	0.187 ± 0.037	0.131 ± 0.036	0.075 ± 0.013	0.058 ± 0.005
BommoOKA_type5	PBS, Dopa, Tyrosinase, BommoOKA_type5	0.165 ± 0.042	0.085 ± 0.012	0.043 ± 0.007	0.042 ± 0.009
BommoOKB_type1	PBS, Dopa, Tyrosinase, BommoOKB_type1	0.165 ± 0.031	0.119 ± 0.011	0.073 ± 0.025	0.058 ± 0.007

**Table 2:** Experimental grouping for tyrosine inhibition rate detection.

Grouping	Sample Background	Negative Control	Reagent Background	Positive Control	Experimental Group
PBS	√	√	√	√	√
Dopa	-	√	-	√	√
Tyrosinase	-	√	√	√	√
β-Arbutin	-	-	-	√	-
Peptides	√	-	-	-	√

## Data Availability

Data is contained within the article.
